# Rapid methicillin resistance diversification in *Staphylococcus epidermidis* colonizing human neonates

**DOI:** 10.1038/s41467-021-26392-8

**Published:** 2021-10-18

**Authors:** Manoshi S. Datta, Idan Yelin, Ori Hochwald, Imad Kassis, Liron Borenstein-Levin, Amir Kugelman, Roy Kishony

**Affiliations:** 1 Faculty of Biology, Technion—Israel Institute of Technology, Haifa, Israel; 2 The Neonatal Intensive Care Unit, Rambam Medical Center, Haifa, Israel; 3 Department of Clinical Microbiology, Rambam Medical Center, Haifa, Israel; 4 Faculty of Computer Science, Technion—Israel Institute of Technology, Haifa, Israel

**Keywords:** Evolutionary genetics, Molecular evolution, Bacterial genetics

## Abstract

Early in life, infants are colonized with multiple bacterial strains whose differences in gene content can have important health consequences. Metagenomics-based approaches have revealed gene content differences between different strains co-colonizing newborns, but less is known about the rate, mechanism, and phenotypic consequences of gene content diversification within strains. Here, focusing on *Staphylococcus epidermidis*, we whole-genome sequence and phenotype more than 600 isolates from newborns. Within days of birth, infants are co-colonized with a highly personalized repertoire of *S. epidermidis* strains, which are spread across the newborn body. Comparing the genomes of multiple isolates of each strain, we find very little evidence of adaptive evolution via single-nucleotide polymorphisms. By contrast, we observe gene content differences even between otherwise genetically identical cells, including variation of the clinically important methicillin resistance gene, *mecA*, suggesting rapid gene gain and loss events at rates higher than point mutations. Mapping the genomic architecture of structural variants by long-read Nanopore sequencing, we find that deleted regions were always flanked by direct repeats, consistent with site-specific recombination. However, we find that even within a single genetic background, recombination occurs at multiple, often non-canonical repeats, leading to the rapid evolution of patient-specific diverse structural variants in the SCC*mec* island and to differences in antibiotic resistance.

## Abstract (web summary)

*Staphylococcus epidermidis* is a widespread early colonizer in the neonatal skin and a cause of hospital-acquired infections. Here, using whole-genome sequencing of 632 cultured *S. epidermidis* isolates derived from premature infants, the authors characterize the spatiotemporally strain-level genomic variability, finding patient-specific colonization signatures and a fast gain and loss of the antibiotic resistance gene *mecA* via the evolution of genotypically diverse structural variants.

## Introduction

Following birth, neonates are colonized with a diverse, highly personalized microbiota, whose early composition influences pediatric health even years later^1–6^. Already during labor and immediately after birth, newborns are exposed to a complex microbiota and are rapidly colonized by bacterial strains originating from their mothers, healthcare professionals, the hospital room environment, and other sources^2,3,7–9^. These initial colonizing strains, which are further influenced by the body environment and antibiotics, can be important in a variety of developmental processes, including maturation of the immune system and pathogen colonization^3,10–13^. Studies of strain diversity in early life have mostly focused on the gut microbiota, where metagenomics analysis revealed long-lasting patient-shared and patient-specific strain colonization^2,14,15^. However, it is unknown how these strains are distributed across the infant body. Furthermore, as unambiguously assembling complete genomes from metagenomic data is inherently challenging, it remains unclear whether and how rapidly gene content and mutational diversity arises within strains during early colonization.

Comparative whole-genome sequencing methods have been tremendously powerful for revealing within-strain evolution of adult chronic bacterial infections, but have not been systematically applied to the neonatal microbiota. In adult chronic infections, comparative whole-genome sequencing methods have uncovered substantial within-strain evolutionary diversification arising on timescales of months or years^16,17^. These studies show how numerous pathogenic populations diversify within human hosts, accumulating mutations that allow them to adapt to challenges presented by the human environment, as well as to overcome antimicrobial treatments^18–24^. Applying similar approaches to pathogen populations on neonates during the first weeks of life can reveal whether within-strain diversity accumulates rapidly in the days or weeks following colonization of a new host, and if so, the underlying mechanisms and phenotypic consequences of such diversification.

The potential for within-strain diversification is of particular importance in *Staphylococcus epidermidis*, a neonatal commensal and opportunistic pathogen with a large pangenome. *S. epidermidis* is a widespread early colonizer in the neonatal skin and gut microbiome, and also a major cause of hospital-acquired infections for premature infants^3,25,26^. Multiple comparative studies of *S. epidermidis* isolated from different patient cohorts demonstrate that infectivity, pathogenicity, and antibiotic resistance varies substantially between lineages due to gene content diversity^27–31^. In particular, genes in the SCC*mec* island, flanked by cassette chromosome recombinase-specific sites, are known to vary in their presence between isolates from even the same MLST group^30,32,33^. The large size of the flexible genome suggests that *S. epidermidis* strains may undergo frequent gain and loss of genes, leading to within-strain genomic diversity on the host.

In this work, we ask how rapidly within-strain diversity arises within otherwise clonal strain populations on newly colonized neonates, using *S. epidermidis* as a model commensal and opportunistic pathogen. We analyzed 632 isolate representatives from nearly clonal strain populations of *S. epidermidis* from the infant skin microbiome. Using a high-throughput, comparative whole-genome sequencing approach, we find that infants are colonized with a highly personalized repertoire of *S. epidermidis* strains, which establish distinct populations across numerous body sites. Within these otherwise clonal *S. epidermidis* populations, patient-specific genomic structural variants differing in antibiotic resistance rapidly arise. In many cases, strains lose resistance to antibiotics through loss of *mecA* via the evolution of genotypically diverse structural variants, suggesting that presence of *mecA* may be an unstable trait on the human body.

## Results

### Spatiotemporal sampling of *S. epidermidis* from nine newborns

**Fig. 1:**
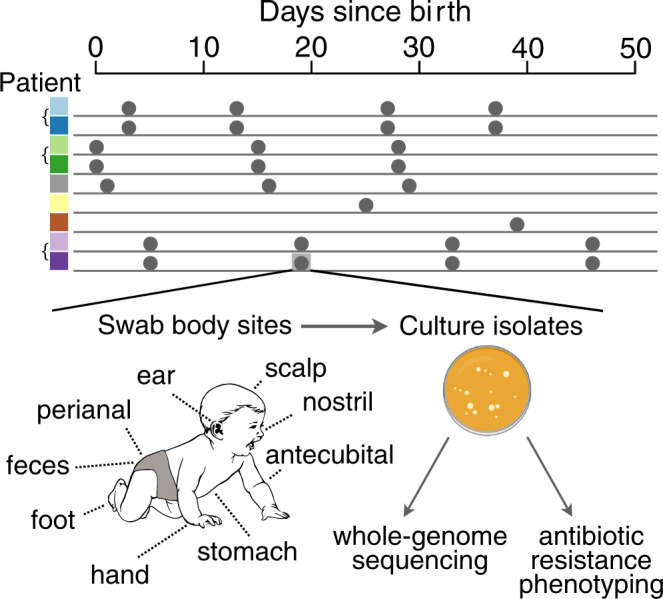
Spatiotemporal sampling of *Staphylococcus epidermidis* from nine newborn infants. Patients are indicated by color, where different shades of the same color correspond to twins. At each of 1–4 time points, infants were swabbed at numerous body sites (not all depicted; see “Methods” section). Swabs were cultured on *Staphylococcus*-selective media (see “Methods” section), from which we collected multiple single colonies. Colonies were harvested and subjected to whole-genome sequencing and antibiotic resistance phenotyping.

To gain insight into de novo colonization and evolution of gene content diversity, we performed extensive spatiotemporal sampling of *S. epidermidis* from the skin microbiomes of nine infants during the first weeks of life. We collected surface skin swabs from nine premature infants, all of whom spent the first 4–7 weeks of life in the neonatal ICU at Rambam Medical Center (Haifa, Israel). These patients included three sets of twins. At each of 1–4 timepoints, we swabbed the neonates at 12–15 skin surface body sites. Note that longitudinal sampling was performed to increase the overall diversity of our sampling effort, rather than providing an explicit time axis for genome evolution. Then, we cultured bacteria from the swabs on *Staphylococcus*-selective media, collected single colonies, and performed whole-genome sequencing and antibiotic resistance phenotyping (Fig. 1 and Supplementary Table 1). Our extensive isolate collection and whole-genome sequencing allows us to identify fine-scale genomic diversification occurring within bacterial populations that could not be observed easily via metagenomic sequencing.

### Rapid “patient-specified” colonization of strains across many body sites

**Fig. 2:**
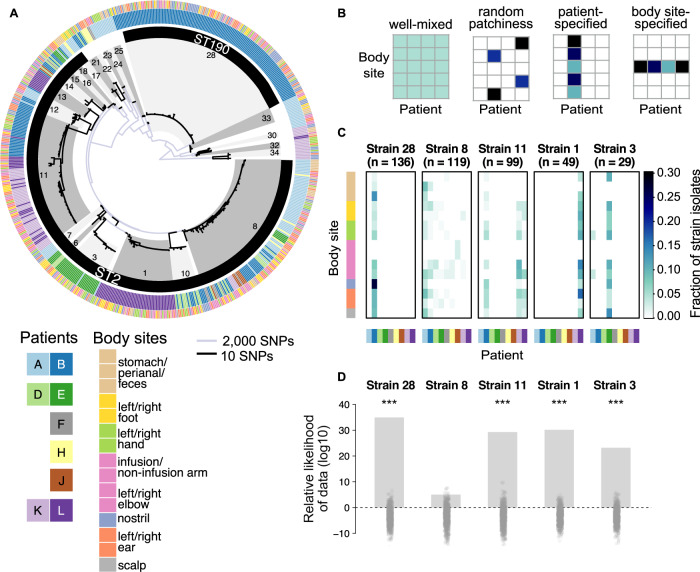
Individual newborns are rapidly colonized by several distinct strains of *S. epidermidis*, which differ substantially in their colonization across patients, but are not body site-specific. **A** Maximum-likelihood phylogeny based on single-nucleotide polymorphisms (SNPs). Individual strains are numbered and designated by alternating grayscale grouping (innermost ring). To depict SNP-level variation within strains (black branches), the scale for within-strain branches has been stretched by 200× compared to between-strain branches (light blue branches). Standard multi-locus sequence typing (MLST) indicates that the majority of strains can be grouped into ST2 or ST190 (black ring). The patient and body site from which isolates were collected are also indicated (outer rings). Twins are indicated by different shades of the same color. **B** Four possible models of strain colonization across patients and body sites. **C** Distributions of the five most sampled strains across all patients. Color scale indicates the fraction of isolates for a given strain that was found on a particular patient/body site combination. **D** Relative log-likelihood (base 10) of strain distributions for each of the five most sampled strains under a patient-only model versus a body site-only logistic regression model. Bars indicate the measured value of the relative log-likelihood for the data. Points indicate the values calculated for randomized strain distributions. ***p-value < 0.001. Source data are provided as a Source Data file.

Analyzing the core genome phylogeny revealed that neonates are colonized with evolutionarily diverged strains of *S. epidermidis*. To characterize genomic diversity across isolates, we constructed a maximum-likelihood tree based on single-nucleotide polymorphisms (SNPs) in genes shared by all isolates (Fig. 2A, see “Methods” section). Furthermore, we assigned each isolate to a multi-locus sequence type (MLST) based on seven conserved genes^34,35^. The majority of isolates were classified as sequence type ST2, which is common in hospital environments and a frequent carrier of antibiotic resistance genes, including methicillin resistance^36,37^. However, even within a sequence type, we observed significant SNP-level diversification into distinct evolutionarily well-separated sub-lineages (“strains”; Fig. 2A and Supplementary Fig. 1, average pairwise between-strain SNP distance: 4208 ± 2352 SNPs). These results suggest that very early in life neonates are independently colonized by multiple coexisting strains.

Individual neonates were colonized by multiple distinct strains of *S. epidermidis*, which differ in their distributions across patients and body sites. Neonates were each colonized by a distinct set of 2–12 strains, with even twins differing in their specific colonizing strains (Fig. 2A; e.g., Strain 28 colonized Patient B, not Patient A; Strain 1 colonizes Patient L, not Patient K). In principle, such differences in strain distributions could be due to stochastic colonization of each site from a shared pool of environmental microbes in the neonatal ICU (“well-mixed”; Fig. 2B, first panel). However, we found that isolates of the same strain were significantly more likely to co-occur within a patient-body site than expected by chance (43.5% of isolate pairs, *p* < 0.001, permutation test; see “Methods” section), suggesting that strains are not uniformly distributed (Supplementary Fig. 3).

Instead, we found that *S. epidermidis* strains tend to colonize infants in a “neonate-specified” manner. We observed that four of the five most sampled strains were strongly biased in their colonization to 1–3 patients, but were detected at numerous body sites on each patient (Fig. 2C). To quantify this bias in colonization patterns, we fit each strain’s distribution to two different logistic regression models: (1) a “neonate-specified” model, in which strain colonization is predicted solely from the neonate’s identity; and (2) a “body site-specified” model, in which strain colonization is predicted solely from the body site (see “Methods” section). Then, we calculated and compared the likelihood of the strain’s distribution under each model. Consistent with the observed bias, the distributions of these four strains (as well as 12 of 33 neonate-colonizing strains sampled) were significantly more likely under the neonate-specified model. Furthermore, no strain was more likely under the site-specified model (Fig. 2D and Supplementary Fig. 4). Of the most widely sampled strains, Strain 8 was the only strain whose distribution was not more likely under the neonate-specified model as compared to the site-specified model. This could be due to the nature of environmental exposure (e.g., coming from a source to which nearly all patients were exposed) or ecological selection (e.g., being able to colonize a wider range of patients).

Together, our results suggest that strain colonization is biased towards particular neonates, potentially due to neonate-specific exposure to particular strains or ecological selection (e.g., by particular antibiotic regimens). Importantly, this biased colonization pattern held across time points; most strains consistently colonized 1–3 patients over time. Furthermore, twins were colonized with distinct strain repertoires even hours after birth and maintained these differences at later time points (Supplementary Fig. 5).

### Analyzing isolate-to-isolate gene content diversification in neonate-colonizing *S. epidermidis* strain populations

Zooming in on within-strain diversity, we asked whether SNP or gene-content diversification occur rapidly enough to lead to functional within-strain diversity on neonates. Analyzing SNP-level diversity, we found little evidence of adaptive evolution via single point mutation; same-strain isolate pairs differed by, at maximum, 13 SNPs (mean: 3.6 ± 3.6 SNPs), with no substantial enrichment for nonsynonymous mutations and no signature of genes mutating more often than expected by chance (Supplementary Fig. 6).

**Fig. 3:**
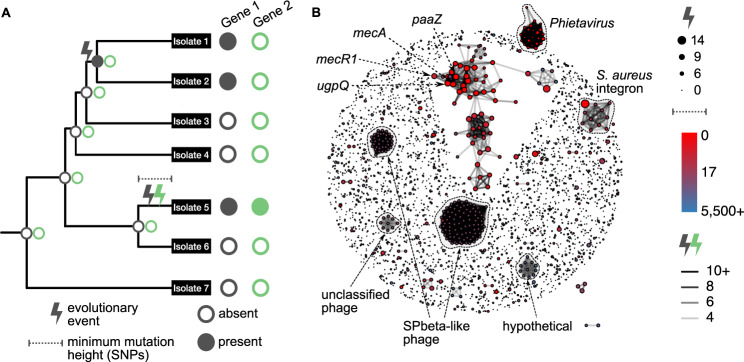
A gene mediating resistance to β-lactam antibiotics (*mecA*) and neighboring genes have evolved recently and repeatedly in multiple *S. epidermidis* lineages colonizing multiple patients. **A** Schematic of inference method to determine how often and how recently gene content evolution has occurred for each gene in the pangenome of this *S. epidermidis* population. **B** Network of pangenome-wide gene gain-loss evolution. Nodes are individual open reading frames (ORFs). The size of the node indicates the number of evolutionary events undergone by that ORF, and the color of the node indicates the height of the most recent mutation (a measure for time since a gene gain or loss event). Edges link genes that evolved concurrently on at least four branches of the population-wide phylogeny. Clusters of genes are annotated by best BLAST hit. Source data are provided as a Source Data file.

In contrast to the slow SNP-level diversification, we identified rapid gene content level evolution. To analyze evolution through gain and loss of genes while minimizing false calls, we devised a three-step hybrid read assembly-mapping process. First, we assembled the genomes of each isolate and constructed a pan-genome containing all open reading frames larger than 30 base pairs and fulfilling our non-redundancy criteria (see “Methods” section). Second, mapping the reads of each isolates to this pan-genome, we determined the presence or absence of each of the pan-genome genes in each isolate (see “Methods” section). Third, we reconstructed the evolutionary gain/loss history for each gene via maximum parsimony and calculated the number of times each gene was gained or lost, as well as how recently evolution occurred (Fig. 3A). As expected, most genes were inferred to have never been gained or lost, as indicated by the high number of small nodes in the network (1,903 genes out of 3,654 analyzed, 76%, consistent with previous estimates of *S. epidermidis* core genome size^27^) (Fig. 3B). Yet, clustering genes into evolutionary co-varying groups (see “Methods” section), we identified 214 genes residing in connected components with more than twenty genes, indicating large groups of genes that evolved concurrently along the same branches of the population-wide phylogeny. Examining the annotations of these genes, we found that these clusters often represent prophage genes or mobile genetic elements (Fig. 3B). Notably, some of these clusters involved antibiotic resistance genes, including *aadK* and *mecA*.

### mecA gain-loss evolution leading to within-strain genome diversification

**Fig. 4:**
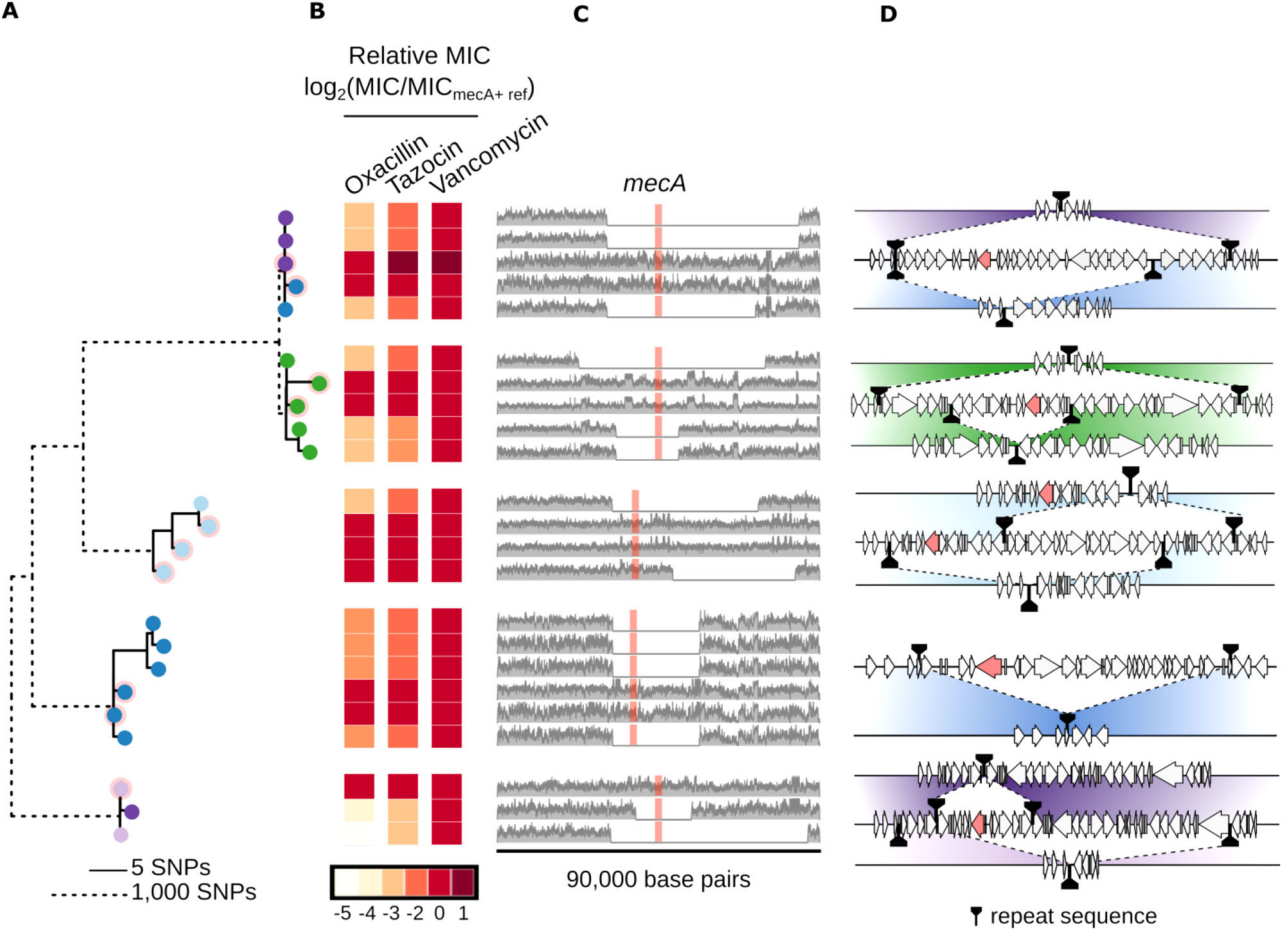
Structural variation in the SCC*mec* island evolved independently in five different *S. epidermidis* strains through site-specific recombination at canonical and non-canonical sites, all leading to functional differences in resistance to β-lactam antibiotics. **A** Maximum-likelihood phylogeny based on single-nucleotide polymorphisms (SNPs) with isolate representatives from strain backgrounds with structural variation in the region containing *mecA*. To depict SNP-level variation within strains (solid line branches), the scale for within-strain branches has been stretched by 200× compared to between-strain branches (dotted line branches). Pink backgrounds denote isolates whose genomes contain *mecA*. **B** MIC of each isolate relative to its closest *mecA*-containing relative for two β-lactam antibiotics (oxacillin and Tazocin) and one non-β-lactam antibiotic (vancomycin). **C** Read coverage across a 90,000-base pair region containing *mecA* (red box). Note that the region of interest is different for each strain background. **D** Putative recombination events leading to structural changes in the SCC*mec* island (*mecA* ORF in red; all other ORFs are gray; repeat sequence based recombination sites in black). Source data are provided as a Source Data file.

Most notably, our analysis revealed that *mecA* belonged to the most dynamic of all the gene clusters, differing in presence even among otherwise clonal isolates, thereby leading to rapid antibiotic resistance diversification. The *mecA* protein is a penicillin-binding protein that confers resistance to a wide range of beta-lactam antibiotics, including methicillin^38,39^. From our pangenome-wide analysis, we found that *mecA* co-evolves with 61 other genes, including genes that are known to be physically closely located to *mecA* on the genome, such as *mecR1* (a regulator of *mecA* expression), *ugpQ*, and *paaZ*. Indeed, our analysis revealed that this cluster comprises the most evolutionarily dynamic genes in the pangenome, as they have evolved often (median = 7 evolutionary events) and very recently (median = 0 SNPs) (Fig. 3B). Namely, even isolates completely identical at the SNP level differ in the presence of *mecA*. Importantly, those isolates lacking *mecA* have lower MICs for beta-lactam antibiotics; on average there was an 8-fold reduction in MIC for oxacillin and tazocin versus no reduction in MIC for vancomycin (Fig. 4A, B). We further tested and confirmed that *mecA* losses did not occur during standard isolation procedures, indicating that laboratory isolation could not explain these losses (none of 950 sampled colonies spontaneously lost oxacillin resistance; see “Methods” section). Altogether, evolution of *mecA* drives rapid within-strain genome divergence, such that even isolates completely identical at the SNP level differ in their levels of resistance to clinically important antibiotics. Additionally, we observed *mecA* evolution in strains colonizing a number of different neonates, including neonates who were or were not treated with beta-lactam antibiotics (Supplementary Table 2).

### mecA evolution shows signatures of promiscuous site-specific recombination

Notably, *mecA* coevolved more frequently with certain genes than with others, even within the connected component, suggesting that multiple independent events underlie *mecA*’s evolution. For each of the five strain backgrounds in which there was isolate-to-isolate variability in the presence of *mecA*, we assembled a high-quality, strain-specific reference genome for a *mecA*-containing isolate, using a combination of short-read and Oxford Nanopore long-read sequencing data. Then, for every isolate representative of a given strain background, we mapped its Illumina short reads to its strain-specific reference genome and calculated the per-base coverage over the entire reference genome (see “Methods” section). To identify regions with extensive gene content evolution, we looked for large regions (>5000 basepairs) with virtually zero per-base coverage in at least one isolate, but high per-base coverage in the reference genome (see “Methods” section Fig. 4C).

Using this computational approach, we found several dramatically different isolate-to-isolate genomic structural variants in the SCC*mec* island. In each strain background, *mecA*-negative isolates typically lacked a large contiguous genomic region (median: 25,085 basepairs) relative to the most closely related *mecA*-positive isolate. This region differed in length in each strain background (Fig. 4C). Furthermore, even within a single clonal background, we identified multiple evolutionary paths leading to structural variation in the SCC*mec* island. For example, of three Strain 1 isolates lacking a *mecA*-containing genomic region, two lack a 53,457-basepair region (isolates denoted by purple dots), while one lacks a 41,288-basepair region (isolate denoted by blue dot). Importantly, these Strain 1 isolates contained no SNPs between them (Fig. 4A, uppermost strain). Extending this analysis to other *S. epidermidis* strains, we found that four out of the five *S. epidermidis* strains had evolved multiple distinct structural variants within otherwise nearly clonal isolate populations (Fig. 4A, C). Overall, our analysis demonstrates that, even within the same strain background, evolution of the SCC*mec* island can occur via multiple different structural genomic changes.

What mechanisms allow SCC*mec* evolution along such distinct evolutionary paths? By assembling reads surrounding these deleted segments, we computationally reconstructed the breakpoint junctions formed upstream and downstream of each evolving region containing *mecA* (see “Methods” section, Fig. 4C). Consistent with recombination, all putative SCC*mec* islands were flanked by homologous sequences (“repeats”) (Fig. 4D and Supplementary Table 3). Notably, repeats were inexact in all cases, consistent with previous reports that repeats need not be identical for SCC*mec* recombination to occur^40^ (Supplementary Table 1). For five of the isolates in which *mecA* was absent (Supplementary Table 1; e.g., ConS_372_0518), repeats contained a short sequence (TATCAT), which has previously been shown to be a conserved recognition sequence for *ccrA*/*ccrB* recombinases mediating SCC*mec* recombination in *S. aureus*^32,33^ (“canonical”). However, for four other isolates, repeats were long homologous regions (100 to >2000 base pairs), with three of them lacking the conserved recognition sequence (“non-canonical”; Supplementary Table 1; e.g., ConS_468_0518). Altogether, the diversity of recombination sites and evolutionary products suggests that the SCC*mec* island can evolve rapidly on the human body through a highly promiscuous recombination process.

## Discussion

In this study, we leverage high-throughput whole-genome sequencing and analysis of over 600 bacterial isolates to characterize the early colonization and accumulation of genomic diversity of *S. epidermidis* on neonates during the first days of life. Analyzing diversity between strains, our study demonstrates that, within the first week of life, neonatal ICU patients are colonized with a unique repertoire of *S. epidermidis* strains. Most strains colonize a small number of patients, but numerous body sites, suggesting a “neonate-specified” model of colonization early in life, with no evidence for body site-specific ecological selection. Zooming in on within-strain diversity, our study reveals gene content diversity within otherwise genetically identical cell populations, suggesting rapid evolution in clinically relevant traits, e.g., antibiotic resistance.

With regard to strain-level colonization, the neonate-specified colonization we observe in our study shows some similarities, but also important differences, compared to colonization patterns observed in full-term infants and in healthy adults. Consistent with our study, full-term infant and adult skin sites are also colonized by multiple *S. epidermidis* strains^27,41–43^, indicating multiple colonization events by genetically distinct founders. However, in contrast with our study, skin-associated bacterial communities of full-term infants and healthy adults often display strong body site-specificity, potentially reflecting diverse microenvironments in adult skin^41,42,44^. The absence of site-specific colonization may come from a number of sources. For example, infant skin may simply be less niche-differentiated than adult skin. Alternatively, given that our study focuses on early colonization events, ecological selection within individual body sites may be difficult to observe.

The dominant pattern of neonate-specified colonization may also reflect the variety of highly individualized clinical treatments preterm infants are subject to early in life, relative to full-term infants or adults. Neonates in our patient cohort differed in their gestational age, birth weight, comorbidities, and antibiotic treatments, all of which could give rise to neonate-specific ecological selective pressures that may dominate at early stages of colonization. Importantly, even twins born vaginally, who underwent the same antibiotic treatment, and who were fed in the same manner differed in their strain repertoire, further emphasizing the neonate-specific nature of *S. epidermidis* strain colonization. However, our study was limited in its ability to identify causal links between clinical treatments and strain colonization. Thus, the role of selection in early colonization, including selection by antibiotics, remains unclear.

Focusing on within strain variation, we found that isolate-to-isolate variability in resistance to beta-lactam antibiotics arises within days or weeks of colonization in otherwise clonal populations of *S. epidermidis* colonizing neonates. Focusing on a key antibiotic resistance locus, the SCCmec island, we found that diversity arises via site-specific recombination involving both canonical and new, non-canonical recombination sites. Importantly, we observe functional differences in gene content even between isolates with otherwise completely identical genomes (no single point mutations differentiating them).

Our finding that individual cells within otherwise clonal populations of *S. epidermidis* may differ in their gene content is consistent with previous studies, which have demonstrated that *S. epidermidis* has an open pan-genome and evolves via horizontal gene transfer from other skin commensals^27^. Furthermore, in line with our model of rapid evolution via promiscuous recombination, the SCCmec island has previously been demonstrated to differ in presence or absence between isolates from the same MLST group^30^, and has been shown to assume diverse genetic architectures across *S. epidermidis* clades^45^. However, our high-throughput whole-genome sequencing procedure allowed us to identify genomic differences in the SCCmec island in isolates with zero SNP distancecolonizing a single patient, suggesting that adaptation via gene gain-loss at this locus occurs at a higher rate than point mutations and may shape evolutionary dynamics during early colonization.

Our study has several notable limitations. While gene content evolution occurs rapidly within *S. epidermidis* populations, we cannot determine where it occurred: either de novo on the patient following colonization, or in the source environment, after which patients were colonized with multiple, nearly clonal genomic variants. Also, our study did not have the statistical power to identify correlations between patient treatment regimens and within-strain diversity, given that each patient was subject to a unique antibiotic treatment regime, as well as differences in birth mode, type of nutrition, or oxygen therapy (see Supplementary Table 2). Thus, the driving forces underlying rapid evolution of the SCCmec island in our study remain unknown. Additionally, our study could not discern the long-term evolutionary patterns within these populations, as strains were only sampled in the first couple of months after birth.

Our discoveries of patient-specified strain repertoire and of gene content variability among clonal isolates within patients raise a number of intriguing hypotheses about the colonization and adaptation of *S. epidermidis* populations during early life. For example, the dramatic evolution of the SCCmec island identified by our computational approach raises the possibility that these phenotypically distinct genomic variants may partition across body sites. Furthermore, our finding that rapid gene-loss events occur via generic, promiscuous recombination events may imply that similar evolutionary diversification occurs in additional species during host colonization, including those colonizing adult hosts. Quantifying these dynamics and analyzing their drivers will be key to harness and engineer bacterial populations for human health.

## Methods

### Patient cohort identification and sampling

Premature infants are at a high risk of developing infections from species of coagulase-negative *Staphylococcus*, particularly *Staphylococcus epidermidis*. Therefore, infants were included with parental consent if (a) they were born before the thirty-seventh week of pregnancy, and (b) their weight at birth was <1500 g. Historically, this population of infants has been widely represented at Rambam Medical Center: roughly 540 infants were hospitalized in 2013, most of them having been born prematurely, and 130 had a birth weight <1500 g. Patients with significant congenital skin disease or congenital malformations of the digestive system were excluded from the study.

Nine premature infants were sampled across multiple body sites on up to four occasions after birth. The first sample was collected within 72 h of birth, and three subsequent samples were collected every 2 weeks thereafter. On each sampling date, swabs were taken from the scalp, each ear canal, back of each hand, back of each foot, inside of the elbow (antecubital fossa), nostril, stomach contents, perianal area, and feces. In most cases, samples were collected using ESwab Liquid Amies Collection and Transport System (VWR #89136-658).

Swabs were stored at Rambam Medical Center at 4 °C for less than 24 h. Subsequently, they were transferred into cryovials containing 1 mL of 25% glycerol and stored at −80 °C until they were used for culture.

### Culturing *S. epidermidis* on selective media

*S. epidermidis* clones were isolated by plating homogenized patient swabs directly on a solid medium consisting of brain-heart infusion (BHI) growth medium supplemented with sodium chloride (7.5% w/v) and agar (1.5% w/v). Cultures were grown on solid media for 24 h at 37 °C. Individual colonies were picked, transferred to 96-well plates containing BHI growth medium with 25% glycerol, and stored at −80 °C for sequencing. We classified approximately 85% of all collected isolates as *S. epidermidis* via analysis of whole genome sequences (see below).

### Whole-genome sequencing of individual S. epidermidis clones

From frozen stocks of individual isolates, 2 μL were plated on solid media containing brain-heart infusion (BHI) growth medium (BD #255003). Cultures were grown for 24 h at 37 °C. After growth, growth spots were collected for whole-genome sequencing.

For each colony, genomic DNA was extracted using the Nucleospin 96 Tissue Kit (Macherey-Nagel #12768552). To aid bacterial lysis, samples were pre-treated with lysozyme (20 mg/mL) and lysostaphin (300 μg/mL) for 30 min at 37 °C before proceeding with DNA extraction. Whole-genome sequencing libraries were prepared from genomic DNA using a miniaturized Nextera XT protocol described previously^46^.

Whole-genome sequencing of individual clones was performed on an Illumina HiSeq 2500 (October 2014, June 2018, and February 2019, paired-end 2 × 150, rapid mode) at the Technion Genome Center (Lorry I. Lokey Interdisciplinary Center for Life Sciences & Engineering).

### Quality filtering of sequenced isolates

Out of 1178 sequenced isolates, 632 were considered for subsequent analyses. These isolates met three criteria:

- We classified the isolate as *Staphylococcus epidermidis* (see below). Out of 1178 isolates sequenced, 990 (84%) were classified as *S. epidermidis*.
- The isolate was sequenced with >20× average genomic coverage, assuming a 2.5-Mb genome. Out of 990 *S. epidermidis* isolates, 813 had >20× average genomic coverage.
- Genomic assembly of isolate reads into contigs was successful and of sufficient length (2 Mb). Out of 813 *S. epidermidis* isolates, 632 had a genomic assembly of sufficient length.

We have detailed the procedures for each of the analyses below.

Raw Illumina paired-end reads were quality filtered and trimmed with Atropos in a three-step procedure^47^. First, low-quality ends were trimmed from reads (*Q* < 15; for both the 5′ and 3′ ends). Second, residual Illumina adapter sequences, including partial adapter sequences, were trimmed from reads. Importantly, matches to adapter sequences were not required to be exact (up to three mismatches allowed). Third, read pairs were filtered based on their error rate (discarded if >15%) calculated from the average *Q* value (typically an overestimate of the true error rate). If either paired-end read fell below the quality threshold, both reads were discarded.

Each isolate was classified as being *S. epidermidis* or non-*S. epidermidis* using Kaiju, a taxonomic classifier for metagenomic reads^48^. Briefly, a random subset of 1000 quality filtered, trimmed Illumina reads was chosen for analysis. Then, each read was assigned to a taxon in the NCBI taxonomy by comparing it to the NCBI RefSeq database, which contains 25 million protein sequences from 7065 complete bacterial and archaeal genomes and 9334 viral genomes. Typically, 20–30% of reads could not be assigned to a species with high confidence. If >50% of reads were classified as “Staphylococcus epidermidis”, the isolate as a whole was considered a representative of that species.

For each sequenced isolate, quality filtered, trimmed reads were assembled into contigs with Unicycler using the default settings^49^. Across all *S. epidermidis* isolates for which the assembly was performed successfully, the assembly had a mean number of bases of 2.6 × 10^6^ across a mean of 118 contigs.

### Assembly of strain-specific reference genomes via MinION sequencing

For subsequent SNP and gene content analyses, we assembled a high-quality reference genome for one isolate from each major population identified in our collection of sequenced isolates. For each isolate, we purified genomic DNA using a NucleoSpin DNA and RNA Purification Kit (Macherey-Nagel #740235). Then, we performed long-read sequencing using a MinION sequencer (Oxford Nanopore Technologies) at the Technion Genome Center (Lorry I. Lokey Interdisciplinary Center for Life Sciences & Engineering). To assemble reference genomes, we used Unicycler^49^ to perform hybrid assemblies, incorporating long-read (Oxford Nanopore Technologies) and short-read (Illumina; see above) sequences. The resulting bacterial genomes were typically circularized and 2.4–2.6 Mb, suggesting that they were virtually complete.

### Isolate MLST classification

To classify a given isolate by multi-locus sequence type, we applied stringMLST to Illumina short-read data for that isolate^35^. We performed k-mer database construction with *k* = 35.

### Characterization of evolution via single-nucleotide polymorphisms

Typically, SNP calling is performed relative to a single reference genome. Instead, we developed a custom iterative procedure, which allowed us to analyze SNPs outside of the core genome, including in genomic regions specific to individual reference genomes. As reference genomes, we used a set of 25 genome assemblies, each of which represents a major strain identified in our isolate collection.

We first ordered these reference genomes according to the following procedure:

- for each isolate, we choose a random subset of 10,000 short reads;
- we map these reads to each reference genome, using blast; and
- we sort the reference genomes by how many reads they successfully recruited (largest to smallest).

Then, for a given isolate, we:

- map quality filtered short reads to the first reference genome, using bowtie v1.1.2;
- map the unrecruited reads to the second reference genome, using bowtie v1.1.2; and
- continue until (a) all reads have been recruited or (b) all available reference genomes have been considered.

Using this procedure, an average of 10% of reads for a given isolate were not recruited to any of the reference genomes considered, indicating that we have the power to detect SNPs across the genome.

For each isolate, we used SAMtools v0.1.19 to generate candidate SNP positions with respect to each reference genome (FQ < −80) . Then, we compiled these positions across all isolates and all reference genomes, and we eliminated positions that were uniform in their base call across all isolates. Positions not represented in the vcf file generated by SAMtools were designated as *N*, and they likely represent positions in the reference genome that are not present in that isolate. For a position positively identified as having more than a single allele across the isolates, the calls of all isolates were inspected to generate a call matrix. For isolates where the call was of low quality (FQ > −50) an “*n*” was written to indicate the uncertainty of the call. Altogether, we identified 32,273 SNPs positions across all reference genomes.

### Tree construction

From these SNPs, we generated a maximum-likelihood tree based on single-nucleotide polymorphisms using IQ-TREE in fast tree search mode (iqtree -s <snp_alignment.fasta> -m GTR+ASC -nt AUTO -fast -o)^50^. As recommended, we optimized a generalized time-reversible (GTR) model of evolution with ascertainment bias correction (ASC), since our SNP alignment did not contain constant sites. The tree was rooted with Staphylococcus capitis subsp. capitis strain AYP1020 (NCBI Reference Sequence: NZ_CP007601.1).

### Strain definition

We grouped isolates by strain based on a fixed cophenetic distance threshold. Using the maximum-likelihood SNP tree generated previously, we calculated the cophenetic distance between every pair of isolates (inter-group dissimilarity at which two isolates are first combined into a single cluster). Then, we identified a cophenetic distance threshold based on the distribution of pairwise distances, in this case, 10^−3.2^ ≈  6 × 10^−4^ (Fig. 2). With this cophenetic distance threshold, the maximum pairwise SNP distance between isolates from the same strain was 3.6 ± 3.6 SNPs (maximum within-strain SNP distance = 13 SNPs), compared to mean between-strain SNP distance of 4208 ± 2352 SNPs.

### Within-strain SNP enrichment analysis

We asked whether specific genes are enriched in within-strain SNPs, suggesting positive selection. We performed this analysis via a three-step process:

- We identified all within-strain SNPs, which may represent within-patient diversification. Out of 32,273 SNPs originally identified, only 223 differentiated isolates within at least one strain. The remainder typically differed between strain lineages, suggesting that they may have occurred before patient colonization.
- Calculate the number of SNPs per open reading frame.
- Repeat calculation for 50 randomized SNP tables. We generated each SNP collection by randomly assigning SNPs to positions from all reference genomes.

### Rejecting “random” colonization model

We tested the hypothesis that strain distribution across patients and body sites is consistent with random colonization from the environmental pool (the strain distribution of all sequenced isolates). As a test statistic, we enumerated all possible isolate pairs, and we calculated the fraction of isolate pairs in which both isolates originate from the same strain. We calculated this statistic for the true isolate collection, as well as for 1000 randomized samples in which the collected isolates were randomized across patients and body sites.

### Determining whether the distribution of each strain across newborns and body sites is patient-specified, or body site-specified via logistic regression

For each strain, we calculated the relative likelihood of its distribution across patients and body sites under two logistic regression models: (1) a patient-only model and (2) a body site-only model (Fig. 2B, C and Supplementary Fig. 4). The patient-only model had the patient names as predictors (nine patients in total), while the body site-only model had the body site names as predictors (15 body sites in total). We fit each of these models to distribution data for a given strain. Then, we calculated the log-likelihood of the data under each model and reported the difference. We repeated the analysis for 1000 randomized samples, in which each strain’s binarized presence or absence was randomized across patients and body sites.

### Characterization of gene presence or absence in individual *S. epidermidis* isolates

To characterize the differences in gene content between *S. epidermidis* isolates, we performed a four-step, hybrid assembly-read mapping procedure. Importantly, this procedure avoids false-negatives in gene content due to poor assembly quality.

First, we assembled a pangenome of open reading frames found across *S. epidermidis* isolates, including low-quality isolates that were not used for subsequent analyses. Starting from each isolate’s assembled genome, we annotated open reading frames using Prokka^51^. Then, we compiled a pangenome of representatives from all annotated ortholog groups using Roary with default settings^52^. Importantly, we did not split paralogs into distinct ortholog groups (-s) to minimize sequence-to-sequence redundancy. We further reduced sequence redundancy in the pangenome by removing : (1) genes with length < 60 base pairs (2× read length; not suitable for our read mapping procedure); and (2) genes with >30 base pairs of homologous sequence shared with another gene in the pangenome. Out of 6725 genes originally identified as part of the pangenome, 5817 genes fulfilled our redundancy criteria. Of these, 5251 genes were found in at least one high-quality isolate genome.

Second, we mapped Illumina short reads for each isolate to the curated *S. epidermidis* pangenome using Bowtie2-2.3.4.1. Importantly, we split reads into 30-base chunks, which facilitated mapping to small open reading frames. Only the single best mapping for each read was considered, and reads with multiple equally valid alignments were discarded. Then, we calculated the per-base read coverage for each gene in the pan-genome using Samtools-1.8 and Bedtools-2.25.0.

Third, we calculated the normalized mean per-base read coverage for each gene. Importantly, we calculated the mean coverage over only the interior of the gene, excluding the 30 base pairs (maximum read length) at each end, as mapping probability decreases linearly over this region. To account for differences in coverage between isolates, we normalized the mean coverage for the gene to the mean, non-zero per-base coverage over the entire genome for the isolate considered.

Fourth, we determined whether each pan-genome gene was present or absent in a given isolate based on its normalized mean per-base read coverage (“coverage”; C). Rather than setting an absolute coverage threshold, we determined thresholds for each gene based on its read coverage statistics. Briefly, we calculated the mean (m) and standard deviation (s) of coverage for genes that were called “present” in isolate genome assemblies, using ORF annotations from assembled genomes as a gold standard for gene presence. Then, we assigned the gene to one of three categories based on the following criteria: (1) “present”: C > m − 2s and there are no gaps in coverage across the length of the gene; (2) “absent”: *C* < 0.001 over the entire length of the gene; (3) “not clear”: *C* > 0.001 and *C* < m − 2s. In cases where a gene’s presence could not be determined unambiguously, its data was considered missing in subsequent analyses.

### Quantifying the minimum number of gene gain-loss events required to explain a gene’s distribution within the sampled *S. epidermidis* population

For each gene, we reconstructed its evolutionary history to determine how often and how recently it was gained or lost. Using the SNP phylogeny, we first assigned each leaf a “state” (presence or absence) based on our previous gene coverage analysis (see above). Leaves for which the state was “not clear” were removed prior to analysis. Then, we inferred the states for each of the tree’s internal nodes using a maximum parsimony algorithm based on Sankoff’s dynamic programming algorithm^53,54^. We assumed that transition costs were equal (a gene gain is as likely as a loss). Finally, for each gene, we calculated how often each gene was gained or lost (total transition cost for the most parsimonious reconstruction) and the recency of evolution (height of the minimum subtree containing nodes that differ in their gene state). Note that this procedure is not meant to identify specific gene gain-loss events precisely, as there can be evolutionary events that are not well captured by our heuristic procedure (e.g., horizontal gene transfer). Instead, we are simply comparing the minimum number of events required to explain each gene’s distribution across the population-wide phylogeny.

### Identification of plasmid vs. chromosomal genes

We sought to distinguish plasmid-associated genes from chromosomal genes, as both may evolve within *S. epidermidis* populations, but via different mechanisms. To identify plasmid-associated genes, we first used isolate genome assemblies to compile a set of putative plasmids, which we defined as small (<500,000 base pairs) circularized contigs. We annotated the open reading frames present in each plasmid. Then, we performed an all-vs-all sequence comparison (BLAST) of genes in the pangenome against these known, annotated plasmid-associated genes. Pangenome genes with significant homology to known plasmid-associated genes were considered plasmid-associated.

### Analysis of spontaneous mecA deletion during culturing on agar plates

Given reports of *mecA* instability in laboratory culture, and the fact that all sequenced isolates were first cultured, we sought to quantify the extent to which individual isolates lose the *mecA* gene during our culturing procedure (described above). To this end, we addressed two questions:

1. Within sequenced colonies, what was the level of variability in the presence of the *mecA* gene?
2. Can a pure *mecA*+ colony lose *mecA* at a sufficient rate to appear *mecA*− after sequencing?

To address these questions, we identified a previously sequenced isolate that was (a) deemed *mecA*+ based on our Illumina sequencing and gene content analysis procedure and (b) from a clonal group in which *mecA* variability across isolates was high. Then, we treated this isolate as a “swab” from which to isolate colonies, performing the same experimental procedure we used to isolate and sequence the colony originally (described below). We cultured the isolate overnight at 37 °C on agar plates containing BHI supplemented with 5% NaCl, using a dilution that allowed us to obtain single colonies. Using a colony picker (Norgren Systems CP7200), we picked nearly 1000 individual colonies from the agar plates and transferred them to a 15% solution of glycerol in PBS. We then stored colonies at −80 °C for at least 24 h. Finally, we thawed cultures completely and pipetted 5 μL of each culture onto an agar plate containing BHI. These cultures grew into large “spots” of roughly 10^8^ cells.

For sequencing, we collected and analyzed DNA from entire spots. Therefore, we assessed the level of variability in the presence of the *mecA* gene within these spots.

To assess the presence of *mecA*− cells within a nominally *mecA*+ spot, we relied on a phenotypic readout: the ability to grow in the presence of oxacillin at concentrations above the MIC of an isolate from the same clonal group, but lacking the *mecA* gene. Specifically, we harvested the cells from a single spot, and transferred them into PBS. To quantify the total number of viable colonies, we plated dilutions of these cultures onto agar plates containing non-selective media (BHI). To quantify the number of *mecA*+ colonies, we plated dilutions of these cultures onto agar plates containing media selective for *mecA*+ cells (BHI supplemented with 0.2 μg/mL of oxacillin). We then used in-house colony counting software to estimate the fraction of *mecA*+ cells out of the total number of cells.

### Quantification of read coverage in the genomic region containing mecA

We sought to identify the location and extent of deletions in the region containing *mecA*. For each of the five strains in which *mecA* presence-absence variability was observed, we first extracted a 90,000-base region containing *mecA* from a fully assembled, strain-specific reference genome (assembled via hybrid short-long read assembly, see above). For each isolate considered from a given strain, we mapped its Illumina reads to the strain-specific, *mecA*-containing region using Bowtie-1.2.2 (bowtie -S -p 4 -a -v 0 –best –strata). We calculated the per-base read coverage for this region using Samtools-1.7 and Bedtools-2.26.0. Then, we normalized the per-base read coverage to the median, non-zero coverage for this region.

### Characterization of canonical and non-canonical recombination sites

For each isolate considered, short reads were mapped to their respective reference genome (bowtie v1.1.2). Deleted loci were identified as regions longer than 5 kb where coverage at all positions was <5 reads while the mean normalized coverage for the ancestral strain was >0.5. To characterize recombination sites, we identified reads which did not map to the reference genome and included 12-base sequences found upstream or downstream of the deleted region in the reference genome. These groups of reads were then assembled separately (velvet v1.2.10) and the resulting contigs mapped to the reference genome using BLAST. Alternatively, where no assembly was generated, a similar procedure was undertaken with 12-base sequences found within the region for which alignment was missing. To validate recombination sites we mapped the reads of the isolate to the hypothetical genome and visually inspected the alignment (igv v2.8.2). Where both approaches failed in identifying the recombination site, the region covering the 5′ edge of the deleted region and stretching 8 kb to each side was BLASTed against the reference genome to identify potential long homologous regions. Finally, the edges of the deleted region, as identified by either BLAST query, with an additional 100 bases to each side, were queried for a set of canonical consensus sequences we identified allowing an additional mismatch.

### Quantification of isolate resistance to oxacillin, tazocin, and vancomycin

For each isolate considered, we quantified the MIC in oxacillin, tazocin, and vancomycin for three single colonies. All colonies were grown in 96-well plates at a range of different drug concentrations for 24 h at 37 °C. The lowest drug concentration at which the background-subtracted OD was less than 0.02 after 24 h was defined as the MIC.

### Ethics committee approval

The study protocol was approved by the ethics committee of Rambam Medical Center, Haifa, Israel. Ethical approval RMB-13-0508. Informed written consent was provided by parents according to IRB guidelines.

### Reporting summary

Further information on research design is available in the Nature Research Reporting Summary linked to this article.

## Supplementary information

Supplementary Information

Reporting Summary

## Data Availability

All single-isolate, whole-genome sequencing data generated in this study have been deposited in the SRA database under project PRJNA757571, Samples SAMN20967709–SAMN20968339, and are available here: https://www.ncbi.nlm.nih.gov/sra/PRJNA757571. Source data are provided as a Source Data file. Source data are provided with this paper.

## Source data

Source Data

## Code availability

Code will be made available upon request.
