# Supplementary material for: Rapid methicillin resistance diversification in Staphylococcus epidermidis colonizing human neonates
Source: Nat Commun. 2021 Oct 18;12:6062. doi: 10.1038/s41467-021-26392-8 (PMC8523572; doi:10.1038/s41467-021-26392-8)
Supplement: Supplementary file 1 — Supplementary Information [file 41467_2021_26392_MOESM1_ESM.pdf]

Supplementary Materials for:
Rapid methicillin resistance diversification in Staphylococcus epidermidis colonizing human neonates

Authors: Manoshi S. Datta[1]†, Idan Yelin[1]†, Ori Hochwald[2], Imad Kassis[3], Liron Borenstein-Levin[2] , Amir Kugelman[2], Roy Kishony[1,4*]

Affiliations:

[1]Technion—Israel Institute of Technology, Faculty of Biology, Haifa, Israel

[2]The Neonatal Intensive Care Unit, Rambam Medical Center, Haifa, Israel

[3]Department of Clinical Microbiology, Rambam Medical Center, Haifa, Israel

[4]Technion—Israel Institute of Technology, Faculty of Computer Science, Haifa, Israel

*To whom correspondence should be addressed: rkishony@technion.ac.il

†Denotes equal contribution

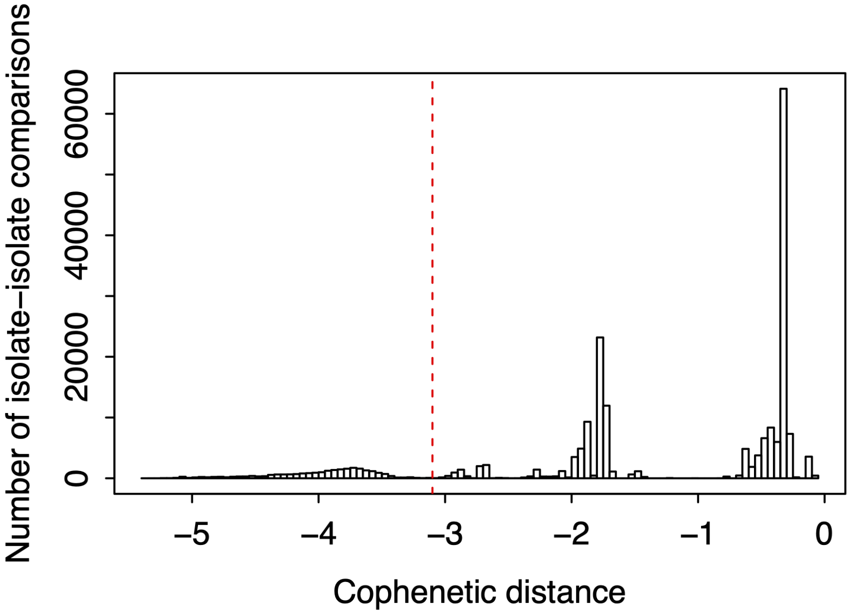

Supplementary Fig. 1. **Defining strains based on SNP threshold.** For all pairs of isolates, the cophenetic distance ("intergroup dissimilarity at which two observations are first combined into a single cluster") was calculated. The cophenetic distance threshold below which two isolates are considered part of the same strain was determined empirically (red dotted line). Source data are provided as a Source Data file.

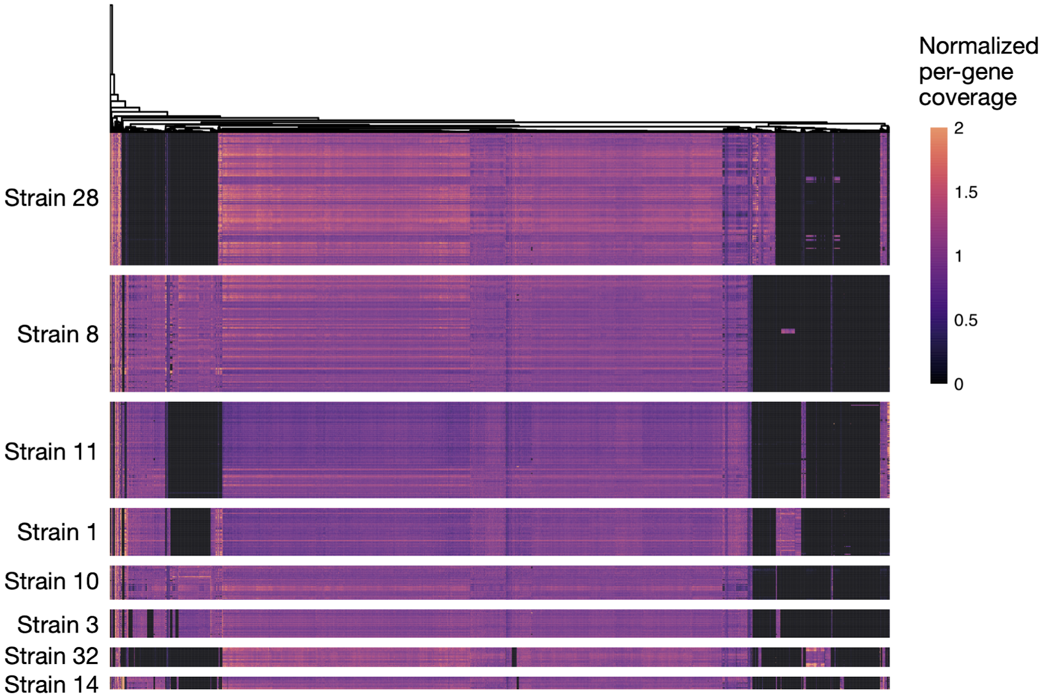

Supplementary Fig. 2. Isolates of the same strains are largely cohesive in their gene content, although there is some within-strain gene content variability. Heat map depicts the normalized per-gene read coverage for all isolates in the top 8 most abundant *S. epidermidis* strains in our isolate collection. Read coverage is normalized per isolate (to the median coverage of all genes present with non-zero coverage) and per gene (by the median coverage across all isolates in which the gene has non-zero coverage). Source data are provided as a Source Data file.

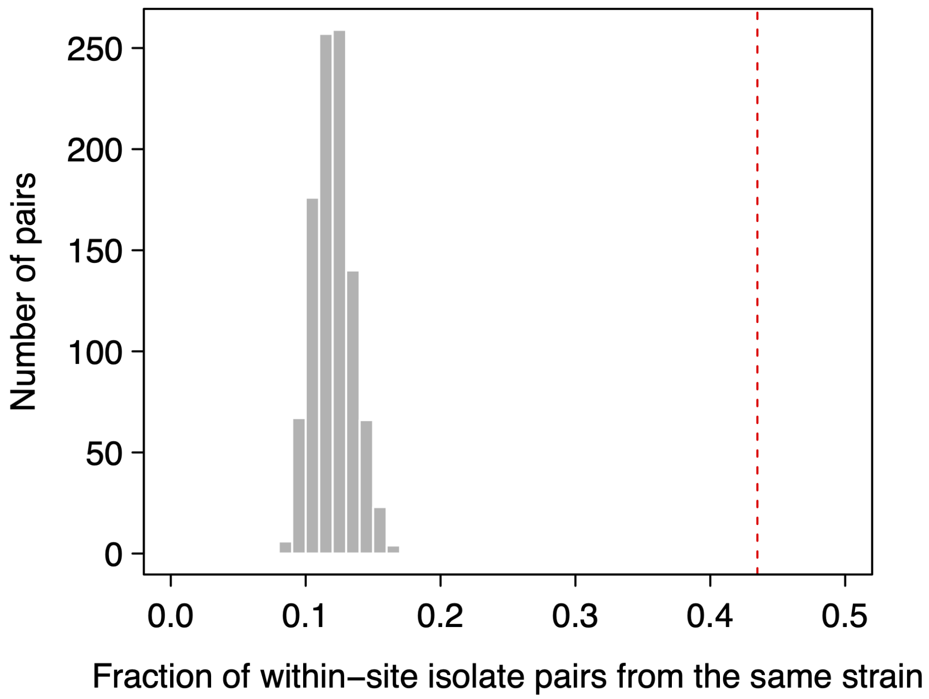

Supplementary Fig. 3. **Strain colonization of patients and body sites is not random.** The fraction of same-strain isolate pairs (out of all possible isolate pairs) was calculated for (1) collected isolate data, and (2) randomized strain distributions across patients and body sites (n = 1,000). Red line indicates the true value based on isolate data collected. Histogram indicates the distribution of values from randomized strain distributions. Source data are provided as a Source Data file.

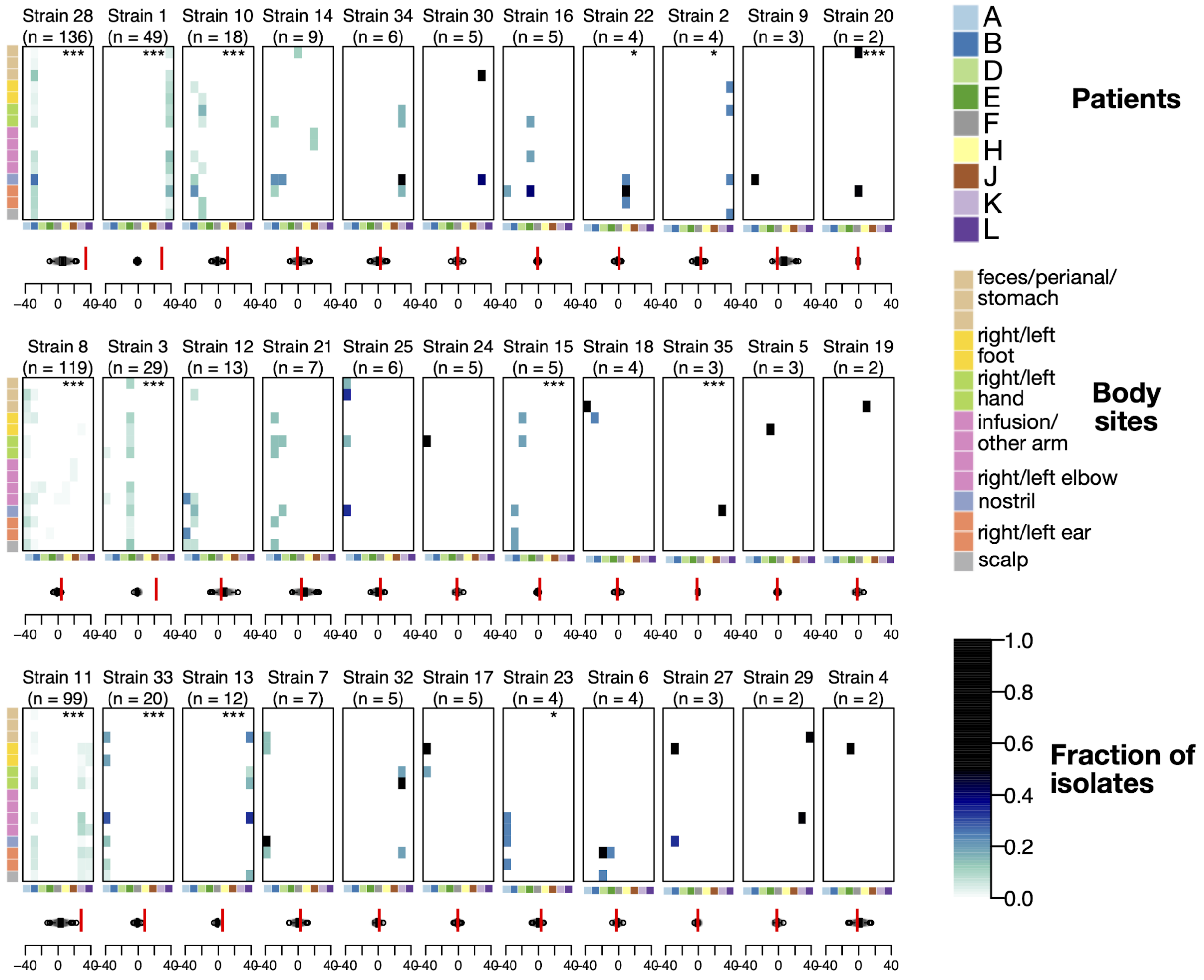

Supplementary Fig. 4. **Strains are largely patient-specified in their colonization, not body site-specified.** Plots show the distributions of all strains across all patients and body sites sampled. Color scale indicates the fraction of isolates for a given strain that was found on a particular patient-body site combination. Bottom bar indicates relative log-likelihood (base 10) of strain distributions under a patient-only model versus a body site-only logistic regression model. Red line indicates actual value. Points indicate the values calculated for randomized strain distributions. Source data are provided as a Source Data file. *** - p-value<0.001.

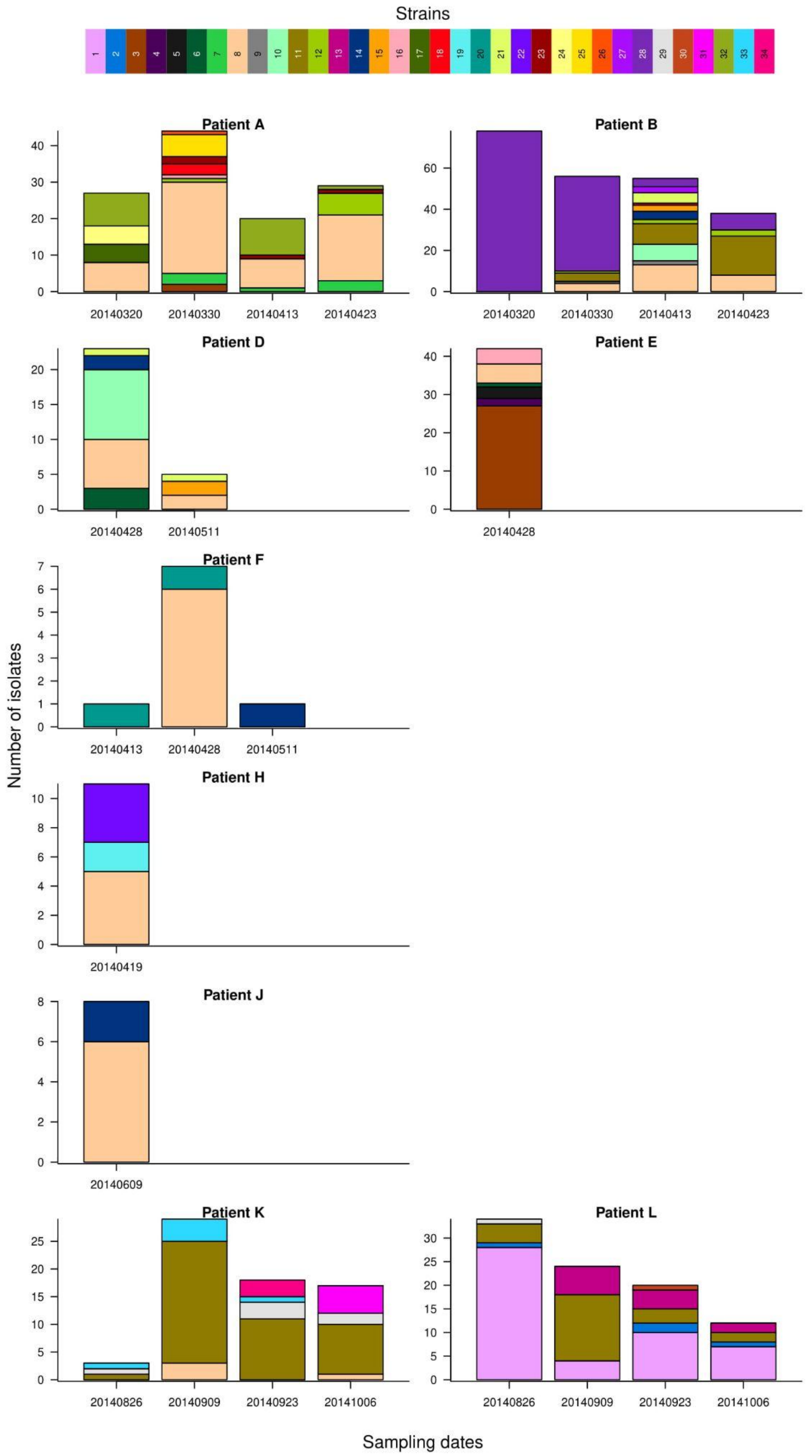

Supplementary Fig. 5. Patients are colonized with multiple distinct strains, which remain biased in their colonization over time. Strain composition for a given patient and time point is indicated as a stacked bar plot, where the total height of the plot indicates the number of isolates sampled. Colors indicate specific strains, as determined by the strain phylogeny (Fig. 2A). Source data are provided as a Source Data file.

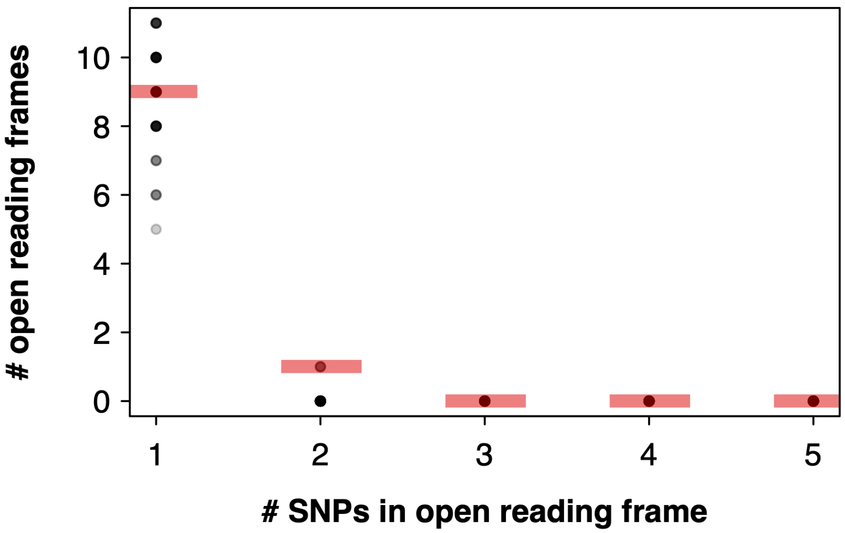

Supplementary Fig. 6. **Number of within-strain SNPs per gene within *S. epidermidis* strains.** Analysis results (red bar) vs. randomized SNP tables (n=50; black dots). Source data are provided as a Source Data file.
