# Supplementary material for: Rapid methicillin resistance diversification in Staphylococcus epidermidis colonizing human neonates
Source: Nat Commun. 2021 Oct 18;12:6062. doi: 10.1038/s41467-021-26392-8 (PMC8523572; doi:10.1038/s41467-021-26392-8)
Supplement: Supplementary file 2 — Reporting Summary [file 41467_2021_26392_MOESM2_ESM.pdf]

nature research

Corresponding author(s): Roy Kishony

Last updated by author(s): Jun 14, 2021

Reporting Summary

Nature Research wishes to improve the reproducibility of the work that we publish. This form provides structure for consistency and transparency in reporting. For further information on Nature Research policies, see our Editorial Policies and the Editorial Policy Checklist.

Statistics

For all statistical analyses, confirm that the following items are present in the figure legend, table legend, main text, or Methods section.

n/a	Confirmed	
☐	☒	The exact sample size (*n*) for each experimental group/condition, given as a discrete number and unit of measurement
☒	☐	A statement on whether measurements were taken from distinct samples or whether the same sample was measured repeatedly
☐	☒	The statistical test(s) used AND whether they are one- or two-sided<br>*Only common tests should be described solely by name; describe more complex techniques in the Methods section.*
☒	☐	A description of all covariates tested
☐	☒	A description of any assumptions or corrections, such as tests of normality and adjustment for multiple comparisons
☐	☒	A full description of the statistical parameters including central tendency (e.g. means) or other basic estimates (e.g. regression coefficient) AND variation (e.g. standard deviation) or associated estimates of uncertainty (e.g. confidence intervals)
☐	☒	For null hypothesis testing, the test statistic (e.g. *F*, *t*, *r*) with confidence intervals, effect sizes, degrees of freedom and *P* value noted<br>*Give P values as exact values whenever suitable.*
☒	☐	For Bayesian analysis, information on the choice of priors and Markov chain Monte Carlo settings
☒	☐	For hierarchical and complex designs, identification of the appropriate level for tests and full reporting of outcomes
☐	☒	Estimates of effect sizes (e.g. Cohen's *d*, Pearson's *r*), indicating how they were calculated

Our web collection on statistics for biologists contains articles on many of the points above.

Software and code

Policy information about availability of computer code

Data collection	No software was used for data collection.
Data analysis	Kaiju v1.6.3 for taxonomic analysis, Unicycler v0.4.6 for genome assembly, stringMLST v0.5.1 for MLST classification, Bowtie2-2.3.4.1 and Bowtie v1.1.2 for short reads alignment, Samtools v0.1.19, Samtools-1.8 and Bedtools-2.25.0 to identify SNPs, IQ-TREE 1.6.12 for phylogenetic trees, Prokka v1.13 to annotate open reading frames, Roary v3.11.2 to generate pangenome, igv v2.8.2 for genome coverage visualization.

For manuscripts utilizing custom algorithms or software that are central to the research but not yet described in published literature, software must be made available to editors and reviewers. We strongly encourage code deposition in a community repository (e.g. GitHub). See the Nature Research guidelines for submitting code & software for further information.

Data

Policy information about availability of data

All manuscripts must include a data availability statement. This statement should provide the following information, where applicable:

- Accession codes, unique identifiers, or web links for publicly available datasets
- A list of figures that have associated raw data
- A description of any restrictions on data availability

All sequencing data that support the findings of this study have been made available through the SRA repository. Accession numbers are detailed in manuscript text.

Field-specific reporting

Please select the one below that is the best fit for your research. If you are not sure, read the appropriate sections before making your selection.

- [x] Life sciences
- [ ] Behavioural & social sciences
- [ ] Ecological, evolutionary & environmental sciences

For a reference copy of the document with all sections, see nature.com/documents/nr-reporting-summary-flat.pdf

Life sciences study design

All studies must disclose on these points even when the disclosure is negative.

Sample size	The sample size of neonates was determined based on inclusion criteria and informed consent.
Data exclusions	No data was excluded.
Replication	We could not replicate this observational study.
Randomization	We included all consenting eligible individuals.
Blinding	No blinding took place.

Reporting for specific materials, systems and methods

We require information from authors about some types of materials, experimental systems and methods used in many studies. Here, indicate whether each material, system or method listed is relevant to your study. If you are not sure if a list item applies to your research, read the appropriate section before selecting a response.

Materials & experimental systems

n/a	Involved in the study	
x		Antibodies
x		Eukaryotic cell lines
x		Palaeontology and archaeology
x		Animals and other organisms
	x	Human research participants
x		Clinical data
x		Dual use research of concern

Methods

n/a	Involved in the study	
x		ChIP-seq
x		Flow cytometry
x		MRI-based neuroimaging

Human research participants

Policy information about studies involving human research participants

Population characteristics	Infants hospitalized in the Rambam hospital NICU were included with parental consent if (a) they were born before the thirty-seventh week of pregnancy, and (b) their weight at birth was <1,500 grams.
Recruitment	Parents of all hospitalized neonates that fit the inclusion criteria were asked for consent.
Ethics oversight	Rambam Medical Center, Haifa, Israel issued IRB RMB-13-0508

Note that full information on the approval of the study protocol must also be provided in the manuscript.
